# Bibliography

**Published:** 1896-12

**Authors:** 


### Bibliography.

A Practical Dental Metallurgy: A Text and Reference Book for Students and Practitioners of Dentistry. By Joseph Dupuy Hodgen, D.D.S., Assistant to the Chair of Dental Chemistry and Metallurgy, University of California, College of Dentistry, etc. San Francisco: The Hicks-Judd Company, 1896.

The author of this volume of three hundred and eight pages says, "In presenting this little volume to the practitioner and student of the dental profession the author does not flatter himself that he is filling a void in such literature, or that a crying need has been felt in the profession for this particular production. . . . The endeavor has not been to furnish a scientific and exhaustive treatise on metallurgy, but rather to present in a clear and practical manner the principles of that subject as the author sees them related and applicable to the every-day wants of the dentist."

While it is true that this branch of dental study has been ably presented in the "American System of Dentistry," and the more practical work of Professor Essig, there is a satisfaction in feeling that the demand for metallurgical knowledge has grown to such an extent as to induce the preparation of this book. The increase of information in this direction by dentists has by no means been in proportion to the age of the profession; indeed, it is questionable whether the metallurgical knowledge of to-day can be compared with that of forty years ago, when every individual practising dentistry was obliged to have some intelligence in this direction. This largely died out during the interregnum between the advent of rubber and the introduction of crown- and bridge-work.

Dr. Hodgen has completed his task carefully, and, it is believed, with a conscientious regard to the wants of the student. It must be judged solely as a text-book, and in this view it should not be subject to the ordinary rules of criticism governing a larger treatise.

Dr. Hodgen is to be congratulated on this his first effort at book-making, and there is no reason why it should not find a place among the text-books of our dental colleges.

NOTES ON THE HISTORY OF ANÆSTHESIA. THE WELLS'S MEMORIAL CELEBRATION AT HARTFORD, 1894. EARLY RECORD OF DENTISTS IN CONNECTICUT. By James McManus, D.D.S. Hartford, 1896.

This history of anæsthesia was prepared at the request of the Connecticut Valley Dental Society, and comes very appropriately before the public at a time when the dispute as to the origin of anæsthesia seems to have been revived by the recent Boston celebration, where all honor was given Dr. W. T. G. Morton. It seems to the writer of this that Dr. McManus's clear statement should carry conviction that Dr. Horace Wells was the true discoverer of anæsthesia, and that whatever merit belonged to Dr. Morton must be found in the introduction of ether through the aid of Dr. C. T. Jackson.

The book gives the history of the celebration in honor of Wells, held at Hartford, December 11, 1894, and also an account of the unveiling of the tablet presented to the city of Hartford, and erected in the building in which the important event occurred. This tablet was designed by E. S. Woods, of Hartford, and cast in bronze by Mr. Mossman, of Chicopee, Mass. An excellent illustration of this is given on the first page.

The record of Connecticut dentistry from 1800 has been a labor of love to Dr. McManus, but it will prove of great historical value to the dentists of that State. The work of the author has been greater than the size of the book would indicate, and is deserving of unstinted praise. If the other States would do a similar work in collecting, it would furnish a basis for the historical student of the future.

A True History of the Discovery of Anæsthesia. A Reply to Mrs. Elizabeth Whitman Morton. By G. Q. Colton. New York, 1896.

This is a pamphlet of decided historical value, and should go with that prepared by Dr. McManus. Dr. Colton is probably the only living witness of this original effort to produce anæsthesia by Dr. Wells, hence anything from his pen on this subject is to be prized.

